# Expression and Role of MicroRNA-663b in Childhood Acute Lymphocytic Leukemia and its Mechanism

**DOI:** 10.1515/med-2019-0101

**Published:** 2019-11-17

**Authors:** Xuehua Liu, Haixia Zhang, Baorong Zhang, Xiaohong Zhang

**Affiliations:** 1 Department of neurotrauma surgery, The First Hospital of Jilin University, No. 71 Xinmin Street, Changchun 130021, China; 2 Pediatric blood and endocrine metabolism nursing platform, The First Hospital of Jilin University, Changchun 130021, China; 3 PICU nursing platform, The First Hospital of Jilin University, Changchun 130021, China

**Keywords:** miR-663b, ALL, CD99, Biological functions, Jurkat cells

## Abstract

Recent studies have shown that microRNAs (miRNAs) play a key role in various malignant tumors. MiR-663b has been found to have important roles in several cancers, however, the role of miR-663b in T cell acute lymphocytic leukemia (T-ALL) remains unclear. Therefore, we speculated that miR-663b might also play a crucial role in the development and process of T-ALL. In the present study, we found that miR-663b was up-regulated in the blood of children with T-ALL and T-ALL cell lines. TargetScan and dual luciferase reporter assay results showed that CD99 was a direct target of miR-663b. In order to further study the biological function of miR-663b in the development of T-ALL and to clarify its potential molecular mechanism, we detected the changes in proliferation, apoptosis, migration, and invasion of T-ALL cell line Jurkat before and after miR-663b inhibitor transfection. We found that miR-663b inhibitor inhibited Jurkat cell proliferation and induced apoptosis. In addition, miR-663b inhibitor repressed Jurkat cell migration and invasion. All these effects of miR-663b inhibitor on Jurkat cells were eliminated by CD99-silencing. These results have provided a new theoretical basis and strategy for the diagnosis and treatment of T-ALL.

## 1 Introduction

Acute lymphoblastic leukemia (ALL) is the most common malignant hematological tumor in children, accounting for 75%-80% of acute childhood leukemia [1]. In recent years, with the application of combination chemotherapy and hematopoietic stem cell transplantation technology, the cure rate of ALL has reached 80%, and the survival rate of leukemia patients has been greatly improved, but 20% to 30% of patients still have bone marrow, testis or central nervous system recurrence [2, 3]. Therefore, we need to strengthen care during treatment, relapse care during treatment, and remission during treatment, and further study the pathogenesis of leukemia, search for new treatment methods, and prognostic markers to improve the quality of life and improve the prognosis of patients.

MicroRNAs (miRNAs) are first revealed in the study of nematode development, and is widely found in eukaryotes. The endogenous non-coding single-stranded small RNA consisting of 19-25 nucleotides and is highly conservative. MiRNAs are often found in the intron region of another gene [4], which specifically binds to the 3’UTR region of the target mRNAs, causing the target gene degradation or inhibiting its translation [5]. In recent years, a number of studies have shown that miRNA changes are closely related to proliferation, metastasis, chemo-sensitivity, diagnosis, and prognosis of many malignant tumors [6, 7, 8]. MiRNAs play a role as tumor suppressor genes or oncogenes in the development of tumors [9, 10, 11, 12, 13, 14, 15].

In 2008, research revealed for the first time the relationship between peripheral blood miRNAs and tumors. For example, in peripheral blood of patients with lymphoma, miR-155, miR-21, and miR-210 levels were significantly higher than those in the control group, and high levels of miR-21 were associated with patient survival [16]. Miyamaeet et al reported that miR-744 is a bio-marker for the diagnosis and prognosis of pancreatic cancer [17]. MiR-19b and miR-183 can be used as potential markers for the diagnosis of lung cancer [18]. A recent study showed that miRNA-100, miRNA-196a, and miRNA-146a play important roles in the development of childhood ALL leukemia and can be used as a bio-diagnostic molecular marker [19].

In recent years, miRNAs have attracted more and more scholars’ attention in leukemia. MiRNAs play a crucial regulatory role in normal hematopoietic function and may be involved in the formation and development of leukemia [20, 21]. It acts as an oncogene or tumor suppressor gene in the formation of leukemia and plays an important role in judging the prognosis of leukemia [22]. At present, great progress has been made in the study of the pathogenesis of miRNAs in ALL. It has been reported that miR-181a-5p promoted proliferation of ALL cells by regulating Wnt signaling pathway, which may be a new target for ALL treatment [23]. MiR-196b/miR-1290 is involved in the anti-tumor effect of resveratrol in acute lymphoblastic leukemia by regulating the expression of IGFBP3 (24). MiR-187-5p regulates the growth and apoptosis of acute lymphoblastic leukemia cells by regulating DKK2 expression [25]. MiR-590 promotes proliferation and invasion of ALL cells by inhibiting the expression of RB1 [26]. Another study showed that miR-101 regulates the progression and chemo-sensitivity of T cell acute lymphoblastic leukemia by targeting Notch1 [27]. In addition, studies have shown that miR-181 is over-expressed in T cell leukemia/lymphoma and is associated with chemo-resistance [28]. These evidences indicated that miRNAs play a very important role in the development of ALL, and its mechanism may be closely related to its regulation of the growth, invasion, and metastasis of ALL cells.

At present, little research has been done on miR-663b in cancer. Cai H et al [29] reported that in pancreatic cancer, miR-663b can be repressed by HOTAIR and exerts anti-cancer effect by targeting IGF2, suggesting that miR-663b and HOTAIR may have important links in the development of cancer. Another study [30] confirmed that miR-663b might be a novel circulating bio-marker for the clinical diagnosis of bladder cancer. However, the expression and role of miR-663b in ALL has not been known so far.

Therefore, this study aimed to investigate the expression and role of miR-663b in ALL, and further to explore its molecular regulation mechanism. It is expected to provide a new strong scientific basis for the diagnosis and treatment of ALL.

## 2 Materials and methods

### 2.1 Clinical specimens

A total of 30 cases of venous bloods from 30 children with T-ALL and 30 healthy volunteers were collected in the First Hospital of Jilin University from June 2015 to June 2017. The present study was approved by the Ethical Committee of The First Hospital of Jilin University, and written informed consent was obtained from each child and the parents or legal guardian of the child.

### 2.2 Cell culture

In this study, human T-ALL cell lines (HPB-ALL, TALL-1, KOPTK1, Jurkat, CCRF-CEM, and Molt16) were purchased from Shanghai Institute of Life Sciences, Chinese Academy of Sciences. ALL cell lines were cultured in RPIM-1640 medium (Gibco, NY, USA) supplemented with 10% fetal bovine serum (FBS) (Gibco, NY, USA). All cells were incubated in a 5 % CO_2_ humidified atmosphere at 37°C.

### 2.3 Cell transfection

Jurkat cells were transfected with inhibitor control (Guangzhou Ribobio Co., Ltd., Guangzhou, China), miR-663b inhibitor (Guangzhou Ribobio Co., Ltd., Guangzhou, China), control-shRNA (Santa Cruz Biotechnology, USA), CD99-shRNA (Santa Cruz Biotechnology, USA), or miR-663b inhibitor+CD99-shRNA for 48 h by using Lipofectamine 2000 (Invitrogen, Carlsbad, CA, USA) according to the manufacturer’s instructions. After transfection, the efficiency of transfection was evaluated by qRT-PCR. Then, MTT assay, trans-well assay, and flow cytometry assay were performed.

### 2.4 QRT-PCR

The total RNA was extracted from blood samples and T-ALL cell lines using TRIzol reagent (Invitrogen, Carlsbad, CA, USA) according to the manufacturer’s instructions. cDNA was synthesized by using a reverse transcription kit (Thermo). cDNAs were analyzed using the SYBR Premix Ex Taq (Takara) under the Applied Biosystems Step One Plus™ Real Time PCR System according to the manufacturer’s protocol. GAPDH or U6 was used as the endogenous control. The primer sequences used were as follows:

CD99, forward 5’-GTGCGGCTAGCACCATGGCCCGCGG-GGCTG-3’;

reverse 5’-CTAGTCTCGAGCTGGTAAGCAATGAAGCTAG-3’;

miR-663b, forward 5’-CGCTAACAGTCTCCAGTC-3’;

reverse 5’-GTGCAGGGTCCGAGGT3’;

U6, forward 5’-GCTTCGGCAGCACATATACTAAAAT-3’;

reverse 5’-CGCTTCACGAATTTGCGTGTCAT-3’;

GAPDH, forward 5’-TGTTGCCATCAATGACCCCTT-3’;

reverse 5’-CTCCACGACGTACTCAGCG-3’;

Bcl-2, forward 5′-TTGGATCAGGGAGTTGGAAG-3′;

reverse 5′-TGTCCCTACCAACCAGAAGG-3′;

Bax, forward 5′-CGTCCACCAAGAAGCTGAGCG-3′;

reverse 5′-CGTCCACCAAGAAGCTGAGCG-3′. The experiments were repeated in triplicate, the relative expression level of genes was calculated using the 2^-ΔΔCT^ method (31).

### 2.5 Western blot assay

The protein was acquired and separated by 12 % SDS-PAGE and then transferred to a PVDF membrane. The membranes were blocked in PBST buffer with 5% non-fat milk for 1.5 h at room temperature. Subsequently, the membrane was incubated with primary antibodies: CD99 (cat no. 16121; 1:1,000; Cell Signaling Technology, Inc., Danvers, MA, USA), Bcl-2 (cat no. Ab196495; 1:1,000; Abcam, USA), Bax (cat no. 14796; 1:1,000; Cell Signaling Technology, Inc.), and β-actin ( cat no. 4970; 1:1,000; Cell Signaling Technology, Inc.) at 4°C overnight, followed by incubation with the secondary antibody (cat no. 7074; 1:2,000; Cell Signaling Technology, Inc.) at room temperature for 2 h. Signals were detected with an ECL system (Merck) according to the manufacturer’s instructions. β-actin was used as the loading control.

### 2.6 MTT assay

Cell viability was detected by MTT assay. Jurkat cells (2.0 × 103/well) were plated into 96-well plates and transfected with inhibitor control, miR-663b inhibitor, or miR-663b inhibitor+CD99-shRNA for 48 h, then 20 μl MTT was added to medium. After incubation for 4 h, the formazan crystals were dissolved with 150 μl DMSO. The absorbance was measured at a wavelength of 490 nm using a microplate reader.

### 2.7 Invasion and migration assays

The 24-well chamber with 8 μm pore size polycarbonate membrane (Corning, Cambridge, MA) was used for migration and invasion of Jurkat cells. To perform cell invasion assay, 12.5 μg matrigel (BD Biosciences) in 50 μl PBS was added to the filter. The cell suspension of the serum-free medium was added to the upper chamber of the transwell insert. The lower chamber was filled with 500 μl 1640 medium supplemented with 20 % FBS. After 24 h incubation at 37°C, the cells on the upper surface of the filter were removed by a cotton swab. The cells that penetrated to the lower surface of the filter were stained with crystal violet and counted under an optical microscope. The difference between cell migration assay and invasion assay was whether to use matrigel.

### 2.8 Dual luciferase reporter assay

TargetScan program (www.targetscan.org/vert_71) was used to predict the targets of miR-663b. The wild type (WT‑CD99) and mutant (MUT‑CD99) 3′‑UTRs of CD99 were cloned into a pmiR‑RB‑ReportTM dual luciferase reporter gene plasmid vector (Guangzhou RiboBio Co., Ltd., Guangzhou, China). For the dual luciferase reporter assay, miR-663b mimic or mimic control, and the wild-type or mutant 3’-UTR of CD99 were co-transfected into Jurkat cells in 24-well plate for 48 h using Lipofectamine 2000 (Invitrogen). Luciferase activity was measured using the Dual-Luciferase Reporter Assay System (Promega, Madison, WI, USA) according to the manufacturer’s instructions. Renilla luciferase was used for normalization. Each sample was performed three times.

### 2.9 Flow cytometry assay

Cell apoptosis was detected by using the Annexin V-FITC/PI apoptosis Detection Kit (Cat no. 70-AP101-100; MultiSciences, Hangzhou, China) according to the manufacturer’s protocol. Cells were harvested, centrifuged, and re-suspended in 100 μl of FITC-binding buffer. Approximately 5 μl of ready-to-use Annexin V-FITC and 5 μl of PI were added to the mixture. Cells were incubated for 30 min in the dark at room temperature. Annexin V-FITC and PI fluorescence were assessed by BD FACSCalibur flow cytometer (BD Technologies).

### 2.10 Statistical analysis

Experiments were shown from three independent experiments in triplicate and representative of all the data. Significance of difference between the two groups was measured by Student’s t test. Comparisons between multiple groups were measured by one-way Analysis of variance (ANOVA) followed by Tukey’s test. All data were analyzed using SPSS 20.0 software (Abbott Laboratories, Chicago, IL) and expressed as the mean ± SD. p<0.05 was considered as significant differences.

## 3 Results

### 3.1 Expression of miR-663b in human T-ALL cell lines and blood of pediatric T-ALL patients

**Figure 1:**
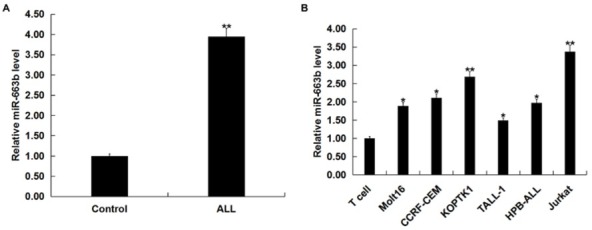
MiR-663b was up-regulated in human T-ALL cell lines and blood of pediatric T-ALL patients. (A) qRT-PCR assay detected the relative expression level of miR-663b in blood of 30 cases pediatric T-ALL patients (ALL) and 30 cases healthy children (Control) (**p<0.01 *vs*. Control). (B) qRT-PCR assay detected the expression level of miR-663b in different human T-ALL cell lines and normal T cells. The data were expressed as the mean ± SD. *,**p<0.05, 0.01 *vs*. T cell.

In order to detect the expression of miR-663b in human T-ALL cell lines and blood of 30 cases of pediatric T-ALL patients, we performed a qRT-PCR assay. The results showed that miR-663b was significantly elevated in the blood of children with T-ALL (Figure 1A) and in different human T-ALL cell lines (Figure 1B); and the highest was in Jurkat cells.

### 3.2 MiR-663b directly targets CD99 in Jurkat cells

**Figure 2:**
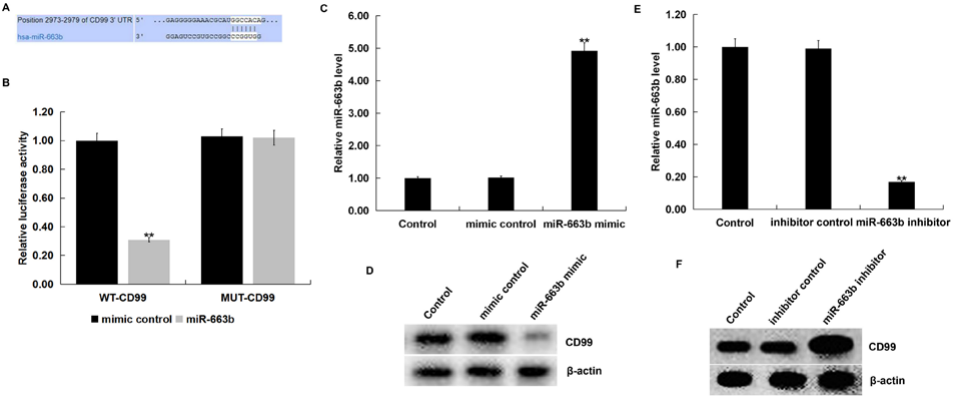
CD99 is the target gene of miR-663b. (A) Binding sites between CD99 and miR-663b were predicted using TargetScan. (B) Dual-luciferase reporter assays were performed to reveal the prediction. **p<0.01 *vs*. mimic control. (C) qRT-PCR assay detected the expression of miR-663b in Jurkat T cells that were transfected with mimic control or miR-663b mimic for 48 h. **p<0.01 *vs*. Control. (D) Western blot assay detected the protein expression of CD99 in Jurkat T cells that were transfected with mimic control or miR-663b mimic for 48 h. (E) qRT-PCR assay detected the expression of miR-663b in Jurkat T cells that were transfected with inhibitor control or miR-663b inhibitor for 48 h. **p<0.01 *vs*. Control. (F) Western blot assay detected the protein expression of CD99 in Jurkat T cells that were transfected with inhibitor control or miR-663b inhibitor for 48 h.

The potential targets of miR-663b were predicted by using TargetScan (Figure 2A). TargetScan revealed the binding sites between miR-663b and the 3’UTR of CD99 mRNA. Furthermore, we performed a luciferase reporter assay to examine whether miR-663b interacted directly with CD99. Dual-luciferase reporter assay revealed that miR-663b mimic significantly decreased the luciferase activity of cells co-transfected with the WT-CD99 and miR-663b mimic, but had no significant effect on the luciferase activity of cells co-transfected with the MUT-CD99 and miR-663b mimic (Figure 2B). Besides, to explore whether miR-663b could regulate CD99 expression in Jurkat cells, Jurkat cells were transfected with miR-663b mimic, miR-663b inhibitor, or their negative controls for 48 h, then the level of miR-663b in Jurkat cells was detected using qRT-PCR and the protein level of CD99 in Jurkat cells was detected using western blot assay. Results showed that compared with the control group, miR-663b mimic significantly enhanced miR-663b level in Jurkat cells (Figure 2C), while decreased CD99 protein expression (Figure 2D). Compared with the control group, the miR-663b inhibitor significantly decreased miR-663b level in Jurkat cells (Figure 2E), while increased CD99 protein expression (Figure 2F). Taken together, these results showed that CD99 was a direct target of miR-663b.

### 3.3 The expression of CD99 in human T-ALL cell lines and blood of pediatric T-ALL patients

**Figure 3:**
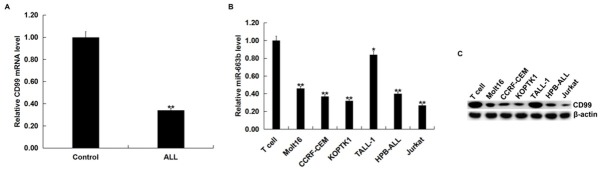
CD99 was down-regulated in human T-ALL cell lines and blood of pediatric T-ALL patients. (A). qRT-PCR assay detected the relative mRNA expression level of CD99 in blood of 30 cases pediatric T-ALL patients (ALL) and 30 cases healthy children (Control) (**p<0.01 vs. Control). (B and C) qRT-PCR assay and western blot assay assay detected the mRNA and protein expression level of CD99 in different human T-ALL cell lines and normal T cells. The data were expressed as the mean ± SD. *,**p<0.05, 0.01 vs. T cell.

In order to detect the expression of CD99 in human T-ALL cell lines and blood in pediatric T-ALL patients, we performed qRT-PCR assay. The results showed that CD99 was significantly decreased in the blood of children with T-ALL (Figure 3A) and in different human T-ALL cell lines (Figure 3B and C); and the lowest was in Jurkat cells.

### 3.4 Effect of miR-663b inhibitor on proliferation, migration and invasion of Jurkat cells

**Figure 4:**
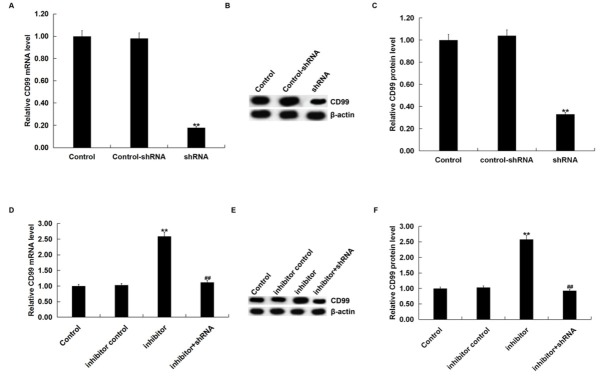
The expression level of miR-663b and CD99 in Jurkat cells after transfection. qRT-PCR assay (A) and western blot assay (B and C) detected the mRNA and protein expression CD99 of in Jurkat cells that were transfected with control-shRNA or CD99-shRNA. qRT-PCR (D) and western blot assay (E and F) detected the mRNA and protein expression of CD99 in Jurkat cells that were transfected with inhibitor control, miR-663b inhibitor or miR-663b inhibitor+CD99-shRNA for 48 h. The data were expressed as the mean ± SD. **p<00.01 vs. Control; ##p<00.01 vs. inhibitor.

To explore the effect miR-663b on Jurkat cell proliferation and apoptosis, we transfected Jurkat T cells with inhibitor control, miR-663b inhibitor, control-shRNA, CD99-shRNA, or miR-663b inhibitor+CD99-shRNA for 48 h. Compared with the control group, CD99-shRNA significantly decreased the mRNA and protein expression of CD99 in Jurkat cells (Figure 4A-C). qRT-PCR analysis and western blot analysis also indicated that compared with the control group, the miR-663b inhibitor significantly enhanced the mRNA and protein expression of CD99 in Jurkat cells (Figure 4D-F), while these effects were eliminated by CD99 silencing in Jurkat cells.

**Figure 5:**
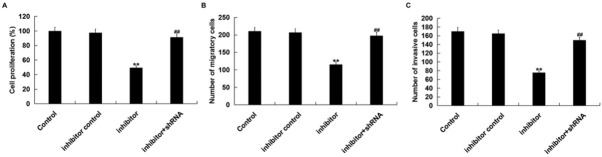
MiR-663b inhibitor inhibited Jurkat cell proliferation, migration and invasion ability. Jurkat cells were transfected with inhibitor control, miR-663b inhibitor or miR-663b inhibitor+CD99-shRNA for 48 h. (A) MTT assay was carried out to determine cell proliferation; (B and C) Transwell assay was carried out to determine cell migration and invasion ability. The data were expressed as the mean ± SD. **p<00.01 vs. Control; ##p<00.01 vs. inhibitor.

In order to shed light on the function of miR-663b in Jurkat cells, we first investigated the effect of the miR-663b inhibitor on the proliferation of Jurkat cells. MTT assay results showed that compared with the control group, the miR-663b inhibitor significantly decreased Jurkat cell proliferation (Figure 5A). Then, we explored the effect of the miR-663b inhibitor on the migration and invasion ability of Jurkat cells. Transwell assay indicated that miR-663b inhibitor significantly reduced Jurkat cell migration and invasion ability (Figure 5B and C). All these effects were eliminated by CD99 silencing.

### 3.5 Effect of miR-663b inhibitor on apoptosis of Jurkat cells

**Figure 6:**
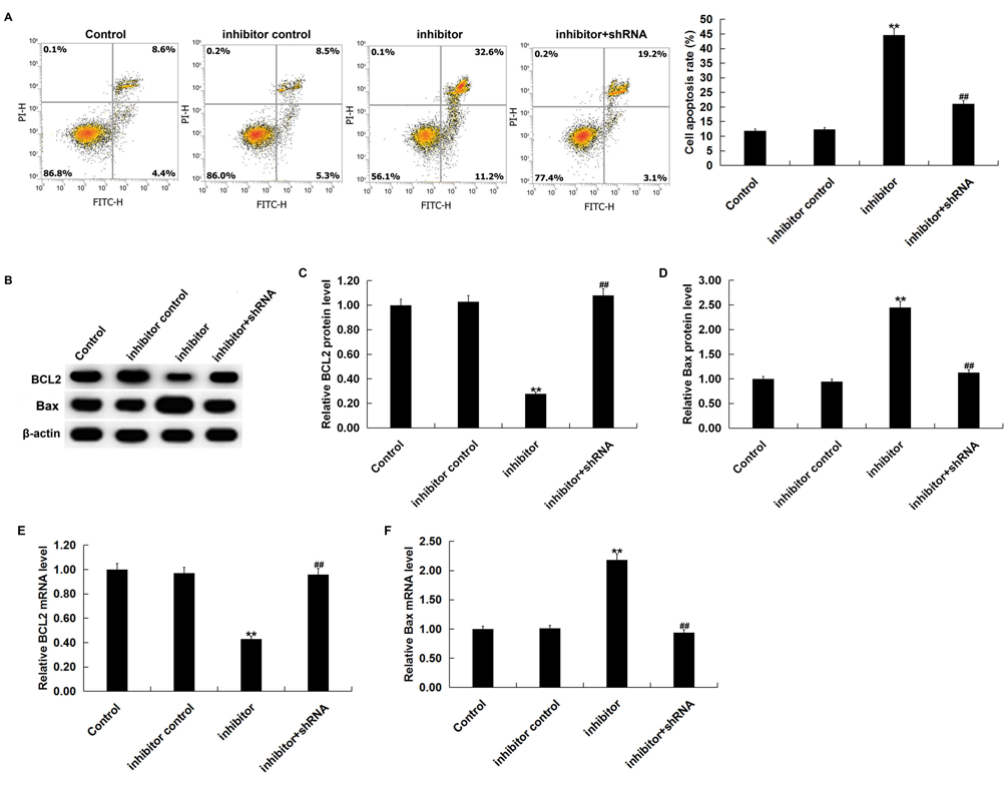
miR-663b inhibitor induced Jurkat cell apoptosis, decreased Bcl-2 expression and increased Bax expression. Jurkat cells were transfected with inhibitor control, miR-663b inhibitor or miR-663b inhibitor+CD99-shRNA for 48 h. (A) Flow cytometry analysis analyzed the percent of apoptotic cells Jurkat cells. (B-D) Western blot assay detected BCL2 and Bax protein expression. (E and F) qRT-PCR detected BCL2 and Bax mRNA expression. The data were expressed as the mean ± SD. **p<00.01 vs. Control; ##p<00.01 vs. inhibitor.

To further determine whether miR-663b could regulate apoptosis, we performed flow cytometry to detect apoptosis, and western blot assay was used to measure apoptosis-related proteins. Flow cytometry analysis showed that miR-663b inhibitor significantly induced apoptosis in Jurkat cells (Figure 6A). Moreover, compared with the control group, the miR-663b inhibitor significantly reduced BCL2 protein and mRNA expression and increased the protein and mRNA expression of Bax in Jurkat cells (Figure 6B-F). All the effects of the miR-663b inhibitor on Jurkat cells were reversed by CD99 silencing. These results indicated that miR-663b inhibitor could induce Jurkat cell apoptosis by targeting CD99.

## 4 Discussion

MiRNA and gene expression profiles have provided special insights into the molecular mechanisms of leukemia [32]. In this study, we found that miR-663b was up-regulated in human T-ALL cell lines and the blood samples of childhood T-ALL patients. Integrative bioinformatics prediction and dual-luciferase reporter assay showed that CD99 was a direct target of miR-663b. Functionally, miR-663b inhibitor inhibited T-ALL cell proliferation and induced apoptosis. In addition, miR-663b inhibitor inhibited T-ALL cell migration and invasion.

Among the many identified miRNAs, miR-663b has been identified to be a novel cancer-associated miRNA. A previous study showed that miR-663b exerted its tumor-suppressive function via targeting insulin-like growth factor 2 in pancreatic cancer [33]. Zhao et al [34] reported that miR-663b was up-regulated in osteosarcoma. MiR-663 was obviously highly expressed in nasopharyngeal carcinoma tissues and nasopharyngeal carcinoma cells [35]. However, the expression of miR-663b in T-ALL has rarely been reported. Herein, we demonstrated that miR-663b was highly expressed in T-ALL cell lines and the blood of T-ALL patients.

To investigate the role of miR-663b in ALL, we performed TargetScan to predict the putative targets of miR-663b, and the results indicated the binding sites between cluster of differentiation 99 (CD99) and miR-663b. CD99 is a transmembrane protein encoded by the MIC2 gene, located in the pseudochromosomal region at the end of the short arm of the X and Y chromosomes. As a salivary mucin type glycoprotein, CD99 is ubiquitously expressed in different types of cells. Functionally, CD99 is involved in the regulation of a variety of cellular events, such as cell adhesion, cell migration, apoptosis and differentiation, and protein phagocytosis [36]. In addition, studies have shown that CD99 is lowly expressed as a tumor suppressor in a variety of malignancies, such as breast cancer, cervical cancer, and lung and gastric cancer [37, 38, 39, 40]. However, CD99 expression and mechanism of action in ALL have been unclear so far. In our study, we confirmed that CD99 was a direct target of miR-663b. And we found that miR-663b negatively regulated CD99 expression in Jurkat cells. And CD99 was significantly enhanced in T-ALL cell lines and the T-ALL patient samples. Therefore, we hypothesized that miR-663b might affect the growth and metastasis of T-ALL cells by regulating the expression of CD99.

Cai et al demonstrated miR-663 could promote cell proliferation in pancreatic cancer cells [33]. MiR-663b down-regulation repressed proliferation and promoted apoptosis in osteosarcoma [34]. Similarly, we found that miR-663 inhibitor inhibited Jurkat cell proliferation, invasion and migration, and induced Jurkat cell apoptosis. It was worth mentioning that all the effects of miR-663b inhibitor on Jurkat cells were significantly reversed by CD99 silencing. However, how CD99, a target of miR-693b, regulates cell proliferation, migration, invasion and apoptosis induction in ALL cells requires further in-depth research.

In conclusion, the findings of the present study suggested miR-663b may be a novel therapeutic target for T-ALL treatment. In addition, patients should be physically and mentally happy during treatment, get quality care, relieve pain, reduce complications, shorten treatment time, and improve the condition. However, this is only a preliminary study of the role of miR-663b in ALL. There is a huge difference between in vitro research and human disease. In order to make the role of miR-663b in ALL more convincing, a lot of experimental research is needed, for example, the role of CD99 alone in ALL cells should be investigated. The relationship between the expression of miR-663b and CD99 and the clinical features of children with ALL needs to be revealed. The effect of miR-663b on other ALL cell lines should be studied. Besides, the role of miR-663b in ALL should be studied in vivo. In the future, we will study these issues.
